# Tackling vitamin A deficiency with biofortified sweetpotato in sub-Saharan Africa

**DOI:** 10.1016/j.gfs.2017.01.004

**Published:** 2017-09

**Authors:** Jan W. Low, Robert O.M. Mwanga, Maria Andrade, Edward Carey, Anna-Marie Ball

**Affiliations:** a International Potato Center, Nairobi, Kenya; b International Potato Center, Kampala, Uganda; c International Potato Center, Maputo, Mozambique; d International Potato Center, Kumasi, Ghana; e International Food Policy Research Institute, Kampala, Uganda

**Keywords:** Orange-fleshed, Sweetpotato, Vitamin A, Biofortification, Integration, Nutrition

## Abstract

Orange-fleshed sweetpotato (OFSP) is a rich plant-based source of beta-carotene, which the body converts into vitamin A. In sub-Saharan Africa, sweetpotato is known as a food security crop but most varieties grown are high dry matter white-fleshed types, lacking beta-carotene. In 1995, researchers recognized the potential of OFSP varieties to address widespread vitamin A deficiency in SSA using an integrated agriculture-nutrition approach. With their partners, they confronted conventional wisdom concerning food-based approaches and institutional barriers, to build the evidence base and breed 42 OFSP varieties adapted to farmer needs and consumer preferences. Subsequently, a multi-partner, multi-donor initiative, launched in 2009, has already reached 2.8 million households. This review summarizes that effort describing how the changing policy environment influenced the process.

## Highlights

• 100 g of orange-fleshed sweetpotato (OFSP) can meet the daily vitamin A needs of a young child.
• Dominant sweetpotato varieties in Africa lack pro-vitamin A; growing OFSP is an easy adjustment.
• Breeding in Africa was requisite to obtain OFSP varieties competitive with local varieties.
• Integrating nutrition education is essential for impact on young child vitamin A status.
• The policy environment drastically changed in 2007, facilitating enhanced investment in OFSP.

## 1 Introduction

### 1.1 Role of sweetpotato

Sweetpotato (*Ipomoea batatas*, L.) came from the Americas to Africa in the 1500s (O'brien, 1972); hence most Africans believe it is indigenous to the continent. Its relative importance varies by sub-region in sub-Saharan Africa (SSA). Currently (2012–2014 average), there are 24.2 million tons of sweetpotato production annually in SSA (Faostat, 2014); over half (54%) is produced in East Africa, 21% in West Africa; 16% in Southern African and 9% in Middle Africa. Conventional wisdom describes its principal role as a food security crop, the crop that is there when the maize fails (Low et al., 2009). This is due to it being relatively drought tolerant once established. On average, it has a higher energy output per area per day (194 mega-joules/ha/day) compared to maize (145 mega-joules/ha/day) (Woolfe, 1992) and the area under sweetpotato has been expanding faster than maize over the past 20 years, albeit from a much lower base (Low et al., 2009). Sweetpotato is vegetatively propagated; therefore planting material (cuttings from vines) is easily shared among farmers. In countries where it is a primary staple (Rwanda, Burundi, Uganda, Malawi), over 80 kg per capita are consumed; where it is a secondary staple, average consumption is 15–40 kg per capita. Table 1 provides production information for 18 sweetpotato producing countries in SSA. Note that China is the world's largest sweetpotato producing country, producing 71 million tons annually. SSA actually has more land under sweetpotato production than China (Table 1) but total production is much lower (24 million tons) as average yields in SSA are 6 t per hectare, compared to 21 t per ha in China.

**Table 1:** Area and Production under Sweetpotato and Population Density in Target Countries in Sub-Saharan Africa and the Sub-Saharan African Region and China (averages over 2012–2014 period).

**Country**	Area (ha)*	**Production (tons)**	**Population density**
			(persons per square km in 2014)*
Angola	151,303	1,257,852	15
Benin	11,324	61,160	90
Burkina Faso	9671	99,368	67
Burundi	85,594	721,175	374
Cote d'Ivoire	25,017	47,935	71
DR Congo	49,053	245,716	33
Ethiopia	51,467	1,889,791	88
Ghana	74,217	135,635	108
Kenya	62,182	784,279	78
Madagascar	143,835	1,115,415	40
Malawi	213,816	3,879,686	147
Mozambique	64,263	2,174,589	31
Nigeria	1,418,056	3,689,696	192
Rwanda	120,934	1,009,105	468
South Africa	20,562	61,035	40
Tanzania	725,301	3,329,726	52
Uganda	452,667	1,841,667	149
Zambia	45,285	167,332	19

**Regions:**			
Sub-Saharan Africa	3,982,844	24,245,681	41.3
China, mainland	3,358,467	71,008,667	145.3

* Source: Area and production data from FAOSTAT, except for Malawi, sourced directly from the Ministry of Agriculture and Food Security. Population density data from the CIA Factbook, accessed at http://www.indexmundi.com/map/?v=21000&r=af&l=en

Most sweetpotato roots in SSA are consumed boiled, steamed or fried. Boiled sweetpotatoes are a common breakfast food. Boiled or fried roots are a common snack, sold in informal markets. For lunch or dinner in rural areas, sweetpotato is often mixed into dishes with beans, cowpeas, coconut milk and/or dark green leaves or served boiled on the side. The degree of leaf consumption as food varies by country.

### 1.2 Why invest in orange-fleshed sweetpotato in SSA?

All types of sweetpotato are good sources of minerals and vitamins, including potassium, phosphorus, vitamins C, K, E, several B vitamins and dietary fiber (Bovell-Benjamin, 2010). The inside of sweetpotato roots (flesh) come in a variety of colors—white, cream, yellow, orange, and purple. Only the orange ones, however, have large amounts of the antioxidant (cancer-fighting) beta-carotene, which the body converts into vitamin A, a vitamin essential for a strong immune system, healthy skin, good vision and eye health (Sommer and West, 1996). The darker the orange color, the more beta-carotene present. Just one small root (100 g) of a medium intensity orange-fleshed sweetpotato (OFSP) variety can meet the daily vitamin A needs of a young child (400 Retinol Activity Equivalents (RAEs)) (Low, 2013). With over 80% of beta-carotene being retained when boiled, few plant foods can match this level (Vimala et al., 2011). Hence, in the West, it now is being promoted as a “superfood” for good health (Eating Well, 2016, Jacobi, 2013, Oliver, 2015, Health Research Staff, 2012). This is reflected in per capita OFSP consumption in the United States rising 80% from 1.9 kg in 2000 to 3.4 kg in 2014 (Johnson et al., 2015).

Globally, 190 million preschool children and 19 million pregnant women are affected by vitamin A deficiency (VAD) (World Health Organization (WHO), 2009). In SAA, more than 40% of children under five suffer from VAD (Black et al., 2013). The main causes of VAD are insufficient intake of vitamin A rich foods, poor absorption of vitamin A or significant loss due to illness (Sommer and West, 1996). In addition to providing adequate vitamin A through dietary means, high dose vitamin A (retinol) capsules can be provided twice yearly or commonly eaten foods (for example, sugar) can be fortified industrially with vitamin A.

In SSA, sweetpotato is a relatively easy crop to grow, and conventional wisdom correctly classifies it as a crop of the poor. Most dominant varieties in SSA are white-fleshed, having no beta-carotene. Women dominate in sweetpotato production in Eastern, Central and Southern Africa. Hence, the use of OFSP to combat VAD makes sense because those most at risk are children in poor households where women are the dominant food preparers and caregivers, and farmers already know how to cultivate sweetpotato (Low, 2013). Moreover, because OFSP is rich in beta-carotene, just 500 m^2^ of a variety producing 10 t per hectare, provides enough vitamin A to meet the annual needs of a family of five.

Because sweetpotato is bulky *and* considered a crop of the poor, levels of consumption per capita in urban areas are often lower than in rural areas (Low et al., 2009). However, few foods are more suited than OFSP to be promoted to counteract the negative effects of the “nutrition transition”. As countries improve economically, typically there is a shift over time from a situation of widespread malnutrition, with inadequate food and nutrients available, to one where there is surplus energy intake and an increase of obesity, often with insufficient consumption of micronutrient rich foods continuing (Masters et al., 2016). The problem is concentrated in urban areas, where physical activity is reduced compared to rural settings. Countries with significant percentages of their population falling in either the under- or over-nourished category are classified as suffering from a “double burden”. Moreover, OFSP not only is an excellent source of quality nutrients, it is a good source of dietary fiber (2.5–3.3 g/100 g). It is digested more slowly than other foods such as bread or potato, with boiled roots having a medium glycemic index (score of 61) and glycemic load (10.7 per 100 g).^1^

### 1.3 Overview

**Fig. 1:**
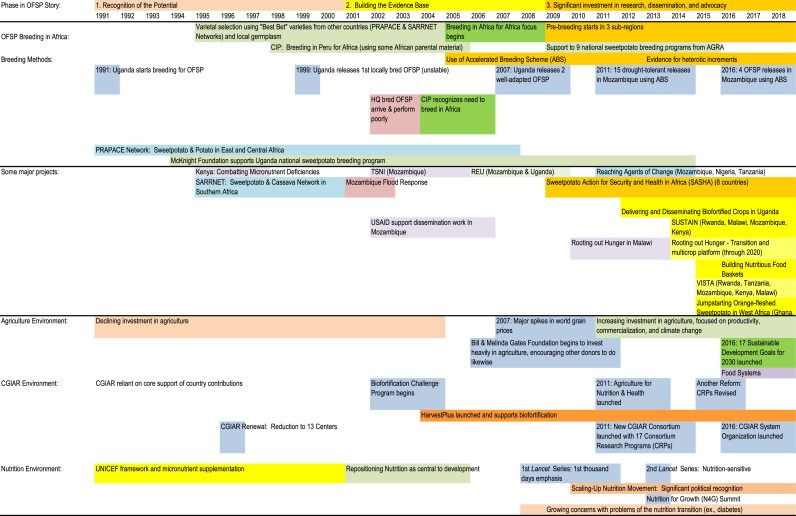
Key phases and events in the development and dissemination of orange-fleshed sweetpotato in sub-Saharan Africa.

The challenges faced in the SSA context are that 1) the dominant varieties currently grown are white- or yellow-fleshed and 2) sweetpotato “seed systems” are underdeveloped. Hence, producers and consumers need to be willing to accept new types of sweetpotato with a distinct visible trait and have timely access to planting material. This paper documents how the promotion and development of OFSP as the lead biofortified crop occurred in SSA from 1995 through mid-2016. Biofortified crops are good sources of energy as well as a key micronutrient (Bouis, 2002). Successful introduction required developing a convincing model for integrating OFSP with nutrition education at the community level, a significant investment in conventional breeding to develop OFSP varieties that were agronomically competitive with dominant local varieties and met local adult consumer taste preferences, and developing viable models of dissemination. Progress to date can be understood in three distinct phases (potential, evidence building, and alignment), defined by the concurrent enabling environments for integrated agriculture-nutrition approaches to flourish (Fig. 1). Biofortified sweetpotato began making significant progress to go to scale only when the policy environment began to recognize agriculture's potential to contribute to nutrition.

## 2 Phase 1: Recognizing the potential (1991–2000)

### 2.1 Confronting the conventional wisdom that Africans will not eat orange

Prior to 1995, the attitude towards OFSP use in SSA by many scientists is captured in the major book on sweetpotato by Woolfe published in 1992: “*Sweet potato cultivars with deep yellow or orange-fleshed roots are unfortunately rejected in many developing countries in favor of white or cream-fleshed types having little or no provitamin A activity*”. Attempts to introduce dark OFSP varieties in several Asian countries failed in the 1980s, not due to the color but due to the moist texture (low dry matter content) of the varieties used. However, conventional wisdom reduced the message to African and Asian consumers do not like OFSP.

In 1995, recognizing the seriousness of the VAD problem in SSA, the International Potato Center (CIP) and the Kenyan Agriculture Research Institute (KARI), began OFSP research as part of a broader effort to develop and test women-based approaches for addressing micro-nutrient deficiencies, a project funded by the International Center for Research on Women.

The research compared OFSP uptake among ten women's groups receiving agricultural extension advice and the new OFSP varieties to ten women's groups receiving the same agricultural intervention *plus* nutrition education (Low et al., 1997). The study found that nutrition education was essential for seeing an increase in the frequency of intake of vitamin A rich foods among young children (Hagenimana et al., 1999). Several OFSP varieties used in the study were found to yield as well or more than dominant local white- or yellow-fleshed varieties. Children liked OFSP varieties that were lower in dry matter content (more moist) than adults, who preferred the floury, higher dry matter content varieties similar to dominant existing varieties. All household members liked the orange color, and the realization emerged that acceptance among adults would be driven by texture and performance, not color. Since young children preferred the moister, lower dry matter OFSP, efforts were targeted to get parents to produce OFSP particularly for their children.

In spite of the positive results among young children, raising funds for food-based approaches during this period was difficult. The global nutrition community was united behind vitamin A capsule supplementation as the magic bullet solution to VAD (Gillespie et al., 2016). The nutrition community cited lack of evidence for food-based approaches, yet the willingness to fund cross-sectoral interventions was low, in part due to each sector operating in isolation.

### 2.2 Breeding in an era of declining resources

Support of official development assistance (ODA) to agriculture plummeted from a high $8 billion dollars (2004 US$) annually in 1984 to $3.4 billion in 2004, only 3.5% of total ODA in 2004. Many large-scale integrated agriculture development efforts had failed in the 1980s, environmental groups saw agriculture as destructive to the environment, and there was increased competition for ODA resources from the social sector. Assistance to Africa declined 50% in the 1990s (The World Bank, 2007). The international agriculture research institutes that comprise the Consultative Group on International Agricultural Research (CGIAR) system were likewise negatively affected. Efforts in many CGIAR centers in the 1990s focused on developing “global public goods”. In the case of sweetpotato, this meant all breeding was done at the International Potato Center (CIP) headquarters in Peru. Limited breeding objectives were set, focused on improved yields, early maturity (3–4 months), and disease resistance. Most OFSP varieties sent to SSA for evaluation during this period were released “best bet” varieties from countries around the world (such as the USA, Peru, Taiwan, and China). While farmers appreciated that many of these varieties were earlier maturing and higher yielding than their local varieties, several were susceptible to high virus pressure. This resulted in low uptake in countries like Uganda and Kenya where pressure was high. In lower virus pressure settlings like Mozambique, several OFSP varieties were promising and in April 1999 the first multi-sectoral stakeholder meeting was organized to promote OFSP utilization.

During this period, sweetpotato varietal selection in SSA was coordinated through two USAID supported networks in East and Central Africa (PRAPACE) and in Southern Africa (SARRNET). Only two SSA countries were actually breeding (i.e. generating crosses between parents), Uganda and South Africa. Notably, breeding including the selection for high beta-carotene started in the Ugandan national program in 1991. The McKnight Foundation provided consistent financial support to the Ugandan program from 1994 through 2014, enabling Uganda to lead OFSP breeding in East and Central Africa. Nine other countries were engaged in varietal selection using “best bets” in limited sites, but overall government and non-government support for most crops was woefully inadequate.

## 3 Phase 2: Building the evidence base (2001–2009)

### 3.1 Proof-of-concept: addressing the doubts of the nutrition community

The nutrition community required evidence of the efficacy and effectiveness of OFSP utilization. Efficacy trials demonstrated the impact on an intervention under “controlled” conditions; whereas effectiveness trials determine whether the intended effect occurs under “real world” conditions.

An OFSP efficacy study conducted among school children (5–10 years old) in South Africa in 2002 measured vitamin A status using the modified-relative-dose response test (Jaarsveld et al., 2005). The treatment group (n=90) consumed 125 g of boiled, mashed OFSP, while the control group (n=90) ate white- fleshed sweet potatoes for 53 school days. The treatment group showed significant improvement in vitamin A liver stores compared to the control group, with the proportion of children in the former group with normal vitamin A status increasing from 78% to 87% after the intervention. There was no significant change in vitamin A liver stores among the control group (decline from 86% at the beginning to 82% at the end).

Further evidence was provided by a community-level intervention in a very resource poor area of Mozambique (the Towards Sustainable Nutrition Improvement project (TSNI)), where VAD prevalence among the children at the beginning of the study was 71%. The intervention consisted of an integrated approach along three intersecting pathways (Low et al., 2007a):

1) Agriculture*: Introduction of a new source of vitamin A and energy, biofortified OFSP:* Vine cuttings are disseminated and lessons provided on crop management and vine conservation.
2) Nutrition: *Demand Creation and Empowerment through Knowledge:* At the village level, principal caregivers, both women and men, are encouraged and enabled to improve infant and young child feeding practices, hygiene practices, and diversify the household diet.
3) Marketing: *Market Development for OFSP Roots and Processed Products:* Generated demand combined with market development stimulates production, enhances producer income and spreads the health benefits of OFSP to a wider population, all of which contribute to farmers’ willingness to retain OFSP and expand production.

In total there were 498 mother-child pairs captured in the 18 month study that were compared to 243 mother-child pairs from “control” areas where no intervention was made.

By the end of the study, 90% of intervention households produced OFSP. Vitamin A intakes among intervention children (n=498) were much higher than those of control children (n=243) (median 426 vs. 56 µg retinol activity equivalents, P<0.001). OFSP contributed 35% to the vitamin A intake of all children in the intervention area and 90% among those who had consumed it the previous day. Serum retinol data were obtained as a proxy for vitamin A status. Controlling for infection/inflammation and other cofounders, a 15% decline in the prevalence of VAD was attributable to the integrated intervention (Low et al., 2007b). OFSP was well accepted and liked by both adults and children. In fact, the color orange proved to be an effective demand creation and marketing tool and became clearly associated with healthy foods.

### 3.2 A development opportunity emerging from disaster

Southern Mozambique suffered from devastating floods in February-March 2000. This resulted in the first experience using OFSP as a disaster response and mitigation tool. The subsequent 18 month project resulted in numerous organizations being trained in OFSP multiplication and dissemination; over 100,000 households received OFSP vines. A demand creation campaign was initiated which included community-based theater, to introduce OFSP to the community before the vines arrived, and the use of promotional materials (hats, cloth worn as skirts, and T-shirts), all in orange with the printed key message of *the sweet that gives health*. This effort demonstrated that demand for OFSP as a health product could be built alongside educating the population about the importance of vitamin A. This is in contrast to demand-led approaches being advocated at the time; the latter tend to reinforce existing practices and behaviors instead of innovation. The success of the project led to investment by USAID and the Government of Mozambique to disseminate OFSP as part of development efforts in other provinces of Mozambique from 2002 through 2006.

### 3.3 Taking the integrated agriculture-nutrition approach to scale

The next step was to investigate whether an OFSP-led, food-based integrated approach could be taken to scale at reasonable cost. Drawing on a modified version of the three pathways outlined above, simultaneous studies led by HarvestPlus^2^ were conducted in Uganda and Mozambique. Known as the Reaching End Users project (REU), two different levels of intensity of extension contact were tested, using extensionists supported by volunteer promoters recruited from the community (HarvestPlus, 2010). The two-year intervention program reached 14,000 households in Mozambique and 10,000 households in Uganda. A randomized controlled trial effectiveness study evaluated the intervention's impact on the intake of OFSP and vitamin A among children 6–35 months and 3–5 years of age and women in both countries (Hotz et al., 2011, Hotz et al., 2012), and on the vitamin A status of young children in Uganda (Hotz et al., 2012).

In Mozambique, 77% of households adopted OFSP compared to 65% in Uganda (de Brauw et al., 2015). In both countries, vitamin A intakes increased significantly among both women and young children, with OFSP contributing 78% of total vitamin A intake among children 6–35 months of age in Mozambique and 53% in Uganda. The less intensive model was just as effective as the more intensive (and more costly) model (de Brauw et al., 2015). Average costs per target beneficiary were $86/household in Mozambique and $56/household in Uganda. In terms of the vitamin A benefit, the intervention in Uganda cost $15–20 USD per disability-adjusted life years (DALYs) saved (Harvestplus, 2010). This amount falls within the “highly cost-effective” category of interventions as defined by the World Health Organization. The wide dissemination of the REU findings provided convincing evidence to the nutrition community that biofortified OFSP could work at scale.

### 3.4 The enabling environment begins to improve

While the agricultural community had begun to recognize the potential contribution of OFSP by the year 2000, the nutrition community to a large extent was not yet supportive, often citing lack of convincing evidence. An interest group spearheaded by the International Potato Center, the Vitamin A for Africa (VITAA) Platform was created in 2001 to raise awareness and serve as a forum for exchange initially among over 70 stakeholders in five SSA countries. A 2001 *ex-ante* study estimated that switching from white-fleshed to orange-fleshed sweetpotato could significantly contribute to reducing VAD in 50 million African children (Low et al., 2001). Around the same time, HarvestPlus, a major program looking at biofortification across several crops, received significant funding (in 2002 and 2004) and the term *biofortification* was coined. HarvestPlus became a significant supporter of OFSP breeding and the VITAA Platform (2002–2008).

During the first half of the decade, the nutrition community, also plagued by low amounts of funding, began to focus on providing evidence that good nutrition was essential for quality human and economic development. In 2008, an influential series of articles on maternal and child nutrition were published in the leading journal, *Lancet.* This resulted in refocusing efforts on community-level interventions to address child stunting, especially among 6–24 month olds and their mothers.

In 2007 the global food price crisis in grains occurred and stimulated a major change in the enabling environment. The vulnerability of import dependent developing countries to surging and unstable prices brought investment in agriculture back onto the agenda among governments and donors in the international community. The Bill & Melinda Gates Foundation became a new and influential donor in agriculture in 2006. Moreover, interest in having agriculture do a better job of addressing nutritional needs began to grow significantly.

### 3.5 Broader recognition of the need to breed in Africa for Africa

Molecular marker work has established that East African farmer varieties (landraces) are distinct from non-African germplasm (Tumwegamire et al., 2011, Gichuki et al., 2003), indicating that Africa has become a secondary center of diversity. East African landraces are notable for high dry matter content (>30%), resistance to local virus strains, vigorous foliage growth and of medium to long maturity (5–7 months).

During Phase 2, the McKnight investment in the Ugandan breeding program paid off, with two moderately virus resistant, good tasting (higher dry matter) OFSP varieties (Kabode and Vita) released in Uganda in 2007 and extensively used in the REU project. These well performing varieties were subsequently released in Kenya (2011) and Kabode in Tanzania (2016). In Mozambique, scientists realized that while “best bet” OFSP varieties yielded well, their vines did not survive the dry season and roots left in the field did not sprout at the onset of the next rains, in contrast to local landraces. In addition, several new OFSP varieties bred in Peru arrived in SSA in 2002 and performed poorly under high virus pressured conditions. By 2005, the obvious need to breed in Africa was in wide acceptance.

Typically, funds to support breeding are hard to raise because traditional breeding methods take 8–9 years from crossing to final release. In 2005, the CIP head of global sweetpotato breeding developed the accelerated breeding scheme (ABS), which uses more sites at earlier stages in breeding, permitting faster selection. This reduced the time from crossing to release to 4–5 years (Grüneberg et al., 2015). In 2005, the Rockefeller Foundation provided four years of support for breeding in Mozambique, which ultimately led to the release of 15 more drought tolerant OFSP varieties in 2011 (Andrade et al., 2016b).

## 4 Phase 3: The stars align (2010–2016)

### 4.1 Major investment breakthrough in synchrony with an enabling environment

The convincing findings from the REU study provided a strong evidence base for further investment in OFSP and the integrated approach as few other models had been examined so rigorously. In 2009, the Bill & Melinda Gates Foundation funded CIP to lead the five-year Sweetpotato Action for Security and Health in Africa (SASHA) project, the largest investment in sweetpotato research ever made in SSA (22.5 million USD). The grant supported the establishment of advanced breeding (population development) programs in 3 sub-regions to address virus resistance, drought tolerance, and quality (a non-sweet sweetpotato), seed system research, and further research on delivery models, including establishing a value chain for developing a commercial OFSP-based processed product in Rwanda and testing a model where pregnant women attending ante-natal care clinics received improved nutrition counseling and vouchers to redeem for OFSP planting material. The SASHA project was renewed for a second five year phase in 2014, with post-harvest research substituting delivery system research.

This core support enabled CIP to launch, along with 26 partners, the Sweetpotato for Profit and Health Initiative (SPHI), a multi-partner, multi-donor initiative with the goal of reaching 10 million households by 2020 in 17 target SSA countries with improved varieties of sweetpotato and their diversified use (Low, 2011). A strong partnership was formed with the Alliance for a Green Revolution (AGRA) support national sweetpotato breeding efforts in 9 SSA countries over the next five years. Moreover, ten other non-governmental and research organizations have joined the SPHI, which holds annual meetings to assess progress.

Concurrently, the Scaling-up Nutrition (SUN) movement emerged. SUN is a platform that encourages countries to commit to a comprehensive set of nutrition targets, calling for heavy investment in community-based nutrition interventions focused on reducing chronic malnutrition (stunting). Irish Aid, DFID (UKAID), and USAID became vocal supporters of nutrition and hunger efforts, with the United Kingdom hosting a global Nutrition for Growth event in 2013, at which several countries made major commitments to invest in nutrition. This included funding from DFID for OFSP dissemination in four countries and a commitment by USAID to include OFSP as an intervention in several of its Feed the Future country level components.

Reviews (Girard et al., 2012, Ruel et al., 2013) noted that OFSP-led interventions were among the few food-based interventions with a good evidence base. The publishing of the second round articles focused on nutrition-sensitive interventions to improve maternal and child well-being in the Lancet in 2013, strengthened the environment for further investment in food-based approaches. During 2012–2013 sub-regional, multi-sectoral planning meetings were held to integrate nutrition into country level Comprehensive African Agriculture Development Programme (CAADP) plans.

### 4.2 Addressing the key bottlenecks to exploiting OFSP's full potential

#### 4.2.1 Adapted OFSP varieties

Having adapted OFSP varieties that consumers like to eat is requisite. In 2005, there were only six OFSP varieties bred in Africa; by 2016 forty-two OFSP varieties had been bred in Africa, with twelve SSA countries actively breeding (Table 2). The major constraint of lack of critical mass involved in sweetpotato breeding has been overcome (Table 2); high dry matter OFSP varieties are available; the accelerated breeding method validated (Andrade et al., 2016b, Andrade et al., 2016a) and the sweetpotato *speedbreeders* are using common protocols and analytic tools as a successful and growing community of practice (Grüneberg et al., 2015). In 2016, 30 breeders working in 14 SSA countries signed a memorandum committing to the principle that at least 50% of the varieties they submit for release will be orange-fleshed.

**Table 2:** Status of sweetpotato breeding in Sub-Saharan Africa.

Year	1995	2005	2016
Number of trained sweetpotato breeders working in SSA (PhD level)	4	8	21
Number of trained sweetpotato breeders working in SSA (MS level)	14	16	13
Number of population development programs	0	0	3
Number of national research programs breeding sweetpotato	2	4	12
Number of national research programs just engaged in varietal selection	0	6	4
Number of released OFSP varieties not bred in SSA	0	13	31
Number of released OFSP varieties bred in SSA	1	6	42
Number of released non-OFSP varieties not bred in SSA	10	41	20
Number of released non-OFSP varieties bred in SSA	8	18	22

#### 4.2.2 Sustainable seed systems

Having adequate quantities of seed of the desired variety at the right time for planting is a challenge for any crop, but particularly for vegetatively propagated crops that have lower multiplication rates than grain crops. Moreover, since most public sector extension services in SSA were curtailed during the 1980s and 1990s, in essence functional public sector distribution systems for sweetpotato were virtually non-existent. Willingness-to-pay for planting material only emerges when there is a high demand for roots in the market, after a widespread drought, or farmers have been convinced that using quality seed results in significantly higher yields. The appropriate seed model is driven by whether the ultimate goal is to have improved nutrition outcomes or improved incomes from root commercialization (Mcewan et al., 2015). Many current approaches seek to build functional linkages between trained vine multipliers closer to the clients and the national programs providing the disease-free, pre-basic starter material. Efforts are underway for eleven national programs to design and implement business plans so pre-basic seed sales continue when project support ends and to lower the cost of pre-basic seed production.

Two important technologies have been developed to improve seed quality and availability. First, in areas with high virus pressure, the use of net tunnels helps train multipliers maintain a stock of disease-free planting material sourced from the national program (Shulte-Geldermann et al., 2012). Second, in areas with dry seasons lasting over four months, the storage of small roots in sand and sprouting in protected beds, known as the Triple S method (Namanda, 2012), has been successfully developed and widely tested in Uganda. On average, one sprouted root generates 40 cuttings for planting ready for the beginning of the rains.

#### 4.2.3 Addressing human resource constraints to enable scaling

Going-to-scale requires having multiple partners engaged, but knowledge about all aspects of sweetpotato production and utilization among many government extension services and non-governmental agencies was low. In response, a ten-day training course on *Everything you ever wanted to know about sweetpotato* was designed, based on a comprehensive set of 13 training of trainers’ modules in four languages (Stathers et al., 2013). Local training institutions implemented the courses with CIP backstopping in Mozambique, Tanzania, Nigeria, Burkina Faso and Ghana from 2012 through 2016. These trainers are then expected to train others. By the end of 2016, over 5000 change agents have been trained.

In response to requests, principally from governments, on how to design and cost an integrated OFSP-nutrition-marketing intervention, a detailed Investment Guide (Stathers et al., 2015a) and complementary Investment Implementation Guide (Stathers et al., 2015b) were developed. Manuals and guides were made available on the Sweetpotato Knowledge Portal (www.sweetpotatoknowledge.org) to encourage broader use.

Finally, through the Reaching Agents of Change project, CIP and Helen Keller International had an unique opportunity to identify and train advocates and develop appropriate advocacy toolkits for OFSP and biofortification in general at the regional level and for three countries: Mozambique, Tanzania, and Nigeria (Mbabu et al., 2015). Advocacy is essential for mainstreaming commitment by governments and regional bodies to integrated agriculture nutrition approaches, such as biofortification. HarvestPlus, CIP and other CGIAR centers engaged in biofortification are intensifying these types of advocacy efforts.

### 4.3 Tackling diversified use – the future growth market for OFSP in SSA

The African continent is rapidly urbanizing. The urban population in Africa is expected to double between 2000 and 2030 (United Nations Population Fund, 2007). Hence, breaking into the urban market is the future growth opportunity for OFSP. In the case of OFSP, there are two entry points: 1) promoting fresh root consumption by changing the image of sweetpotato as a poor person's food to OFSP being a health food for all classes of consumers, and 2) integrating OFSP as an ingredient in processed foods such as bakery products and juices and promoting fried forms of OFSP. Research has demonstrated that the fat in fried OFSP products makes the beta-carotene more bio-accessible than the beta-carotene in baked products (Tumuhimbise et al., 2009). Efforts underway to set standards with the *Codex Alimentarus* will assist the consumer in identifying processed products that are biofortified as such standards define minimum levels of micronutrient content for a product to be considered to be biofortified and enable standardized product labeling (Codex Alimentarus Commission, 2013).

Research efforts to date have successfully demonstrated in Rwanda that steamed and mashed OFSP (purée) can replace significant percentages (35–50%) of wheat flour in baked products (Ndirigwe et al., 2015). A four month shelf-storable (using locally available preservatives), vacuum-packed purée that is less expensive than wheat flour has been developed that should facilitate adoption by the processing industry and is currently being tested with a purée factory in Kenya (Bocher et al., in press). Since many countries import significant amounts of wheat flour, OFSP purée substitution appeals to farmers, processors, and policy makers. To broaden sweetpotato use, the breeding platform in West Africa is focusing on developing non-sweet varieties that will expand their potential use as a staple starch for fresh and processed food markets (Baafi et al., 2016).

The other underdeveloped market with high potential is more extensive use of vines and non-commercial roots in animal feeds. The high protein content of leaves (16% crude protein) makes them an ideal dairy and pig feed. A technique for making silage from vines and roots has been adapted from approaches used in Asia (Lukuyu et al., 2012).

## 5 The challenge ahead

### 5.1 Reaching the 2020 goal

Since launching the SPHI in 2009, four organizations have been tracking the number of beneficiary households receiving improved sweetpotato varieties through their various projects: CIP, HarvestPlus, Farm Concern, and Helen Keller International. As of September 2016, 2.89 million households had been provided planting material of improved varieties in 14 of the 17 target countries. Studies to assess adoption in several countries are underway for dissemination projects completing initial four to five year phases. CIP is seeking funding for a major adoption study effort for 2019, to evaluate the degree of OFSP uptake during the SPHI.

Reaching the remaining 7.2 million will require continued resource mobilization among the donor community but also getting sweetpotato use and dissemination better integrated into country, donor and non-governmental organization mandates and cross-cutting CGIAR research programs. Advocacy efforts at the regional level are focusing on biofortification as the entry point, with OFSP being a crop ready to be expanded. Country level integration needs to occur both at the policy and planning level, so that activities are budgeted.

In addition, special efforts are required for national agricultural sample surveys to collect data on the OFSP types separately from other flesh colors to enable cost-effective monitoring of uptake at scale. To date, this is only occurring in Mozambique where 2015 results show that 32% of sweetpotato produced in Mozambique is orange-fleshed (Ministério, 2015). OFSP messaging also needs to be integrated into national nutrition training tools.

### 5.2 Building on emerging opportunities

To date, emphasis has been on introducing OFSP into areas where sweetpotato is being grown. However, recent experience introducing OFSP into the Tigray region of Ethiopia, where sweetpotato was unknown before, has been quite positive (Jogo et al., 2014). With appropriate water management systems, OFSP clearly has the potential to expand into more semi-arid regions where its dual purpose potential for feed and food is appreciated by communities with sizable livestock assets.

It is also important to influence the parents of tomorrow. OFSP has been successfully integrated on a pilot basis into a school feeding program in Osun State, Nigeria, creating a stable root market for farmers and improving vitamin A intakes of primary school children. In Uganda, efforts are underway to integrate OFSP production and utilization as part of the standard agricultural curriculum in primary school.

However, continued investment in OFSP will depend on its ability to fit into the ever changing policy environment as external policies drive investment decisions. Its multiple roles in the food system as a staple, an emergency response food, a vegetable, feed, an ingredient in processed products or a rotation crop will enhance the likelihood of its use for different desired outcomes (Table 3). In 2016, 17 new sustainable development goals for 2030 were announced. Hunger elimination remains a key goal and good health and well-being is the third goal. Experts argue for placing more emphasis on nourishing populations in ways that can be sustained environmentally and economically, than just on productivity increases in key foodstuffs (Haddad et al., 2016). Early maturing OFSP varieties integrate easily into many food systems facing changing climatic conditions because it produces quickly (3–4 months) within a broad temperature range, can produce on marginal soils, and as a rotation crop with grains, legumes or vegetables, helps manage pest and disease incidence.

**Table 3:** Roles of sweetpotato and expected influence on desired outcomes in the coming decade.

	Roles of Sweetpotato (SEVFIR)*					
	Staple	Emergency	Vegetable	Feed	Ingredient/	Rotation
**Desired Outcomes**		Response			Processed Products	Crop
Food Security	Increase	Same	Increase	Same	Increase	Increase
Nutrition and Health	Increase	**Increase**	**Increase**	Same	Increase	Increase
Income Generation	Same	NA	Increase	**Increase**	**Increase**	Same
Diversified Use	Same	Increase	Increase	Increase	Increase	Same
Climate Change Mitigation	**Increase**	Increase	Increase	Increase	Increase	Increase
Sustainable Food Systems	Increase	NA	Increase	Increase	Increase	**Increase**

* Bold type indicates that a desired outcome is expected to be a major driver underlying increased use.

There is no doubt that the transformation opportunities for sweetpotato into processed products will only continue to expand, the pace depending on the price of sweetpotato relative to other ingredients. Improving productivity through better varieties, more efficient seed systems, and improved crop management and post-harvest practices will help lower overall price and drive expanded industrial use. Current South African commercial sweetpotato yields of 50–60 t per hectare and average yields in China (21 t per hectare) reveal the large yield potential yet to be tapped, as average yields elsewhere in SSA range from 4 to 13 t per hectare under rain fed conditions. Consumer awareness of nutritional benefits will enhance demand and willingness to pay. This will require continued innovative efforts with public and private entities to promote more nutritious foods.

## Footnotes

1 Glycemic Index (GI) measures how much a carbohydrate will raise blood sugar levels compared to glucose (GI=100). A high GI food has a GI of 70 or above and a low GI 55 or less. Glycemic load (GL) takes both the GI and the amount of carbohydrate in a particular food into account. A high GL is over 20 points; a low GL less than 10 points.

2 HarvestPlus is a program coordinating biofortification activities in partnership with many organizations under the CGIAR research program on Agriculture for Health (Bouis and Saltzman, this issue).
